# TNF Drives Monocyte Dysfunction with Age and Results in Impaired Anti-pneumococcal Immunity

**DOI:** 10.1371/journal.ppat.1005368

**Published:** 2016-01-14

**Authors:** Alicja Puchta, Avee Naidoo, Chris P. Verschoor, Dessi Loukov, Netusha Thevaranjan, Talveer S. Mandur, Phuong-son Nguyen, Manel Jordana, Mark Loeb, Zhou Xing, Lester Kobzik, Maggie J. Larché, Dawn M. E. Bowdish

**Affiliations:** 1 Department of Pathology and Molecular Medicine, McMaster University, Hamilton, Canada; 2 McMaster Immunology Research Centre, McMaster University, Hamilton, Canada; 3 Michael G. DeGroote Institute for Infectious Disease Research, McMaster University, Hamilton, Canada; 4 Department of Environmental Health, Harvard School of Public Health, Boston, Massachusetts, United States of America; 5 Clinical Epidemiology and Biostatistics, McMaster University, Hamilton, Canada; 6 Department of Medicine, McMaster University, Hamilton, Canada; University of Toronto, CANADA

## Abstract

Monocyte phenotype and output changes with age, but why this occurs and how it impacts anti-bacterial immunity are not clear. We found that, in both humans and mice, circulating monocyte phenotype and function was altered with age due to increasing levels of TNF in the circulation that occur as part of the aging process. Ly6C^+^ monocytes from old (18–22 mo) mice and CD14^+^CD16^+^ intermediate/inflammatory monocytes from older adults also contributed to this “age-associated inflammation” as they produced more of the inflammatory cytokines IL6 and TNF in the steady state and when stimulated with bacterial products. Using an aged mouse model of pneumococcal colonization we found that chronic exposure to TNF with age altered the maturity of circulating monocytes, as measured by F4/80 expression, and this decrease in monocyte maturation was directly linked to susceptibility to infection. Ly6C^+^ monocytes from old mice had higher levels of CCR2 expression, which promoted premature egress from the bone marrow when challenged with *Streptococcus pneumoniae*. Although Ly6C^+^ monocyte recruitment and TNF levels in the blood and nasopharnyx were higher in old mice during *S*. *pneumoniae* colonization, bacterial clearance was impaired. Counterintuitively, elevated TNF and excessive monocyte recruitment in old mice contributed to impaired anti-pneumococcal immunity since bacterial clearance was improved upon pharmacological reduction of TNF or Ly6C^+^ monocytes, which were the major producers of TNF. Thus, with age TNF impairs inflammatory monocyte development, function and promotes premature egress, which contribute to systemic inflammation and is ultimately detrimental to anti-pneumococcal immunity.

## Author Summary

As we age, levels of inflammatory cytokines in the blood and tissues increase. Although this appears to be an inevitable part of aging, it ultimately contributes to declining health. Epidemiological studies indicate that older adults with higher than age-average levels of inflammatory cytokines are at increased risk of acquiring, becoming hospitalized with and dying of *Streptococcus pneumoniae* pneumonia but how age-associated inflammation increased susceptibility to was not entirely clear. We demonstrate that the increase in the inflammatory cytokine TNF that occurs with age cause monocytes to leave the bone marrow prematurely and these immature monocytes produce more inflammatory cytokines when stimulated with bacterial products, thus further increasing levels of inflammatory cytokines in the blood. Furthermore, although old mice have higher levels of these inflammatory monocytes arriving at the site of *S*. *pneumoniae*, they are not able to clear the bacteria. By pharmacologically or genetically removing the inflammatory cytokine TNF or reducing the number of inflammatory monocytes we were able to restore antibacterial immunity in aged mice. Thus we demonstrate that monocytes are both influenced by and contributors to age-associated inflammation and that chronic exposure to age-associated inflammation increases susceptibility to *S*. *pneumoniae* due to altering monocyte maturity and function.

## Introduction

Monocyte phenotype and function change with age but whether these changes contribute to susceptibility to infectious disease is unclear. In mice, monocytes can be subdivided based on their expression of the Ly6C antigen into Ly6C^high^ (Ly6C^high^, CCR2^high^, CX3CR1^low^) and Ly6C^low^ (Ly6C^low^, CCR2^low^, CX3CR1^high^) monocytes [1,2]. In humans, the functional equivalents are CD14^++^CD16^-/+^ and CD14^+^CD16^++^ monocytes, respectively [1,3]. Ly6C^high^ monocyte output from the bone marrow to the blood increases in a CCR2-dependent manner early during infection [4,5], and they become the dominant monocyte subtype in the blood, preferentially homing to sites of inflammation[6]. Ly6C^high^ monocytes produce high levels of inflammatory cytokines[4,5,7]; thus, they are often called “inflammatory monocytes”.

In the elderly, numbers of circulating CD14^++^CD16^+^ and CD14^++^CD16^-^ monocytes, are significantly higher[8]. CD14^++^CD16^+^ monocytes derived from elderly individuals are more senescent (i.e. have shorter telomeres) than other monocyte subsets and produce more pro-inflammatory cytokines (IL6, TNF, IL1β, IL12p70) and have higher levels of some chemokine receptors (e.g. CCR2, CCR5, CCR7, CX3CR1) [9,10]. Due to their ability to produce large amounts of pro-inflammatory cytokines, Ly6C^high^ monocytes contribute to the pathology of several models of chronic inflammation [11,12,13,14,15,16,17]. During chronic inflammatory conditions, the number of circulating Ly6C^high^ monocytes increase progressively[18] and their ablation is an effective strategy for decreasing pathology [16,17,19,20]. Whether Ly6C^high^ monocytes contribute to chronic age-associated inflammation and increased susceptibility to infection is not known and is the focus of this study.

Aging is accompanied by an increase in the levels of pro-inflammatory cytokines such as tumour necrosis factor (TNF) and interleukins 1β (IL1β) and 6 (IL6) in the serum and tissues, a phenomenon that has been termed “inflamm-aging”[reviewed in[21,22]]. This age-associated, systemic state of chronic, low-grade inflammation (defined as “para-inflammation” by Medzhitov[23])is well-documented although its cellular source has yet to be definitively identified. Adipose tissue[24], CD4^+^ T cells or macrophages[25,26] have all been proposed to contribute. Increases in serum cytokines (particularly IL6 and TNF) are generally thought to be a pathological consequence of aging, as they correlate with risk of classical “diseases of age” such as dementia[27], stroke[28], cardiovascular disease[29] as well as frailty[30,31] and all-cause mortality[32,33]. Conversely, lower than average levels of age-associated inflammation predict good health in age[34]. Furthermore, most chronic inflammatory conditions are characterized by increased numbers of CD14^++^CD16^+^ and/or CD14^++^CD16^-^ monocytes [35,36,37,38,39,40,41]. Herein, we investigate the role of monocytes, which are known to be critical mediators of chronic inflammation, contribute to age-associated inflammation.

Inflamm-aging contributes to susceptibility to infectious disease, and particularly pneumonia, which is a major cause of death in the elderly[42]. Susceptibility to pneumonia correlates with increased levels of IL6 and TNF before an infection [43,44,45]. When young mice are infused with TNF, they become as susceptible to experimental infection with *Streptococcus pneumoniae* as old mice[46]. Using a mouse model of pneumococcal colonization, we investigated whether changes in monocyte phenotype adversely affect host defense towards *S*. *pneumoniae*. We show that with age that there is an in increase in circulating Ly6C^+^ monocytes during the steady state due to increased expression of CCR2. Using heterochronic bone marrow chimeras, we demonstrate that the aging microenvironment, rather than intrinsic changes in myeloid progenitors, drives changes in monocyte phenotype, including decreased expression of F4/80 (a marker of maturity), and increased expression of CCR2 (required for monocyte mobilization). We demonstrate that age-associated increases in TNF are the driving factor behind changes in monocyte phenotype, as TNF deficiency or treatment with anti-TNF antibodies normalizes expression of CCR2 on Ly6C^+^ monocytes. Decreased CCR2 expression results in decreased numbers of monocytes in the circulation and reduced production of TNF and IL6. Finally, we demonstrate that, although TNF levels and the recruitment of Ly6C^+^ monocytes are increased in old mice during nasopharyngeal *S*. *pneumoniae* colonization, this, counterintuitively, results in diminished bacterial clearance.

To our knowledge, this is the first mechanistic study that investigates the role of Ly6C^+^ monocytes as central mediators of inflamm-aging and demonstrates that TNF is a key contributor to age-associated defects in myeloid phenotype and anti-bacterial function. These data indicate that Ly6C^+^monocyte frequency and increased production of pro-inflammatory cytokines contributes to both age-associated inflammation and declining anti-bacterial immunity.

## Results

### Ly6C^+^ monocytes increase with age in the blood and bone marrow but are phenotypically and functionally different

**Fig 1:**
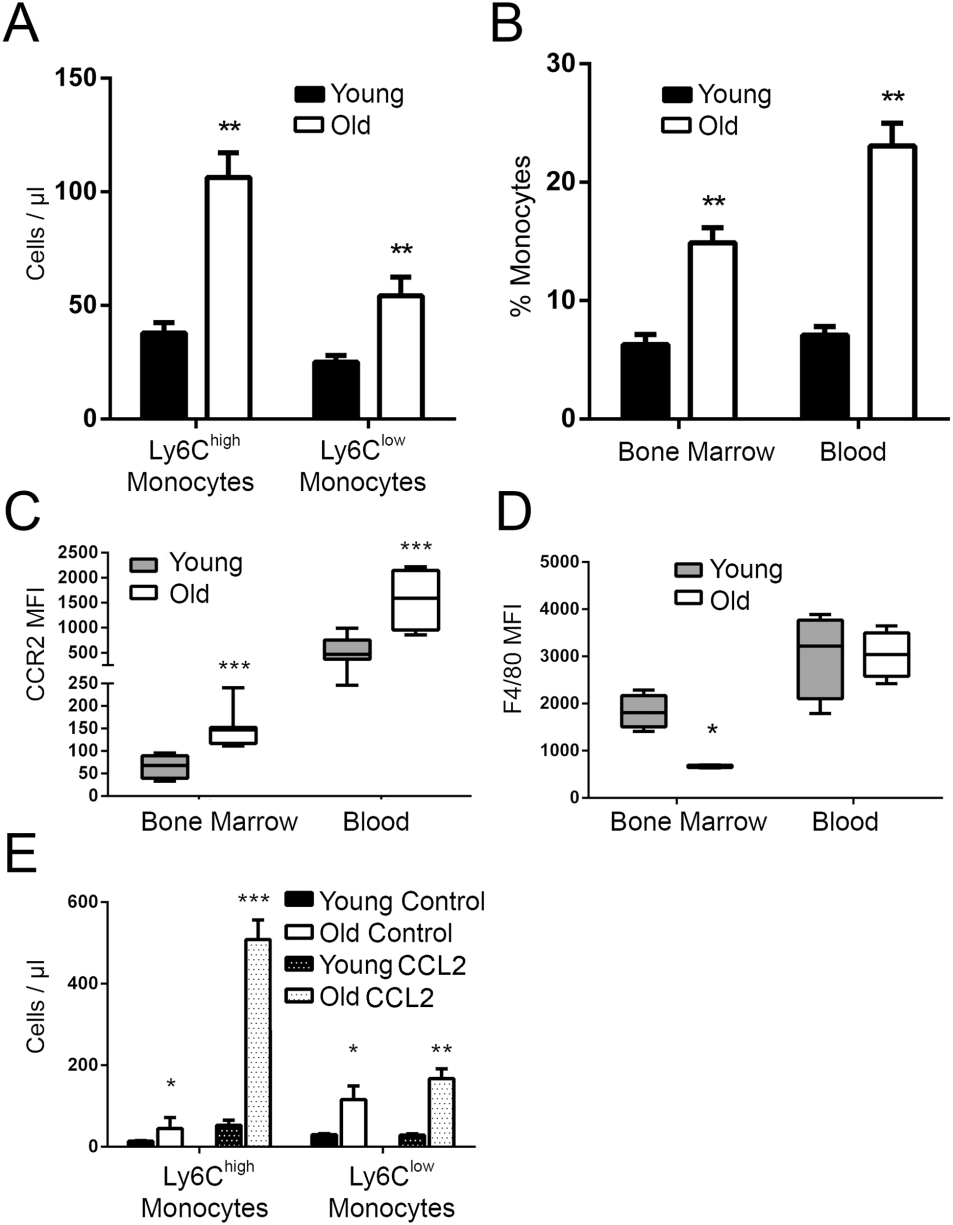
Ly6C^high^ monocytes are increased with age, express more CCR2 and less F4/80. (A) Total numbers of Ly6C^high^ and Ly6C^low^ monocytes were quantitated in the blood of old (18–22 mo) WT C57Bl6/J mice and compared to that from young (10–14 wk) mice. The data represent the mean (± SEM) of 6 mice. (B) Analysis of the Ly6C^high^ monocytes as a percentage of CD45^+^ cells in the blood and bone marrow of young and old mice (± SEM; *n* = 6). (C) CCR2 expression on Ly6C^high^ monocytes in the bone marrow and blood of old mice is higher than young controls as determined by flow cytometry (*n* = 6–8). (D) The mean expression of the macrophage maturity marker, F4/80, on Ly6C^high^ monocytes in the bone marrow and blood of young and old mice (*n* = 6–8). (E) Cells recruited to the peritoneum were quantitated 4 hours after administration of 100 nM CCL2. The recruitment of Ly6C^high^ and Ly6C^low^ monocytes was greater in old mice (± SEM; *n* = 5). Statistical significance was determined by two-tailed Mann-Whitney-Wilcoxon test or two-way ANOVA with Fisher's LSD post-test where appropriate. * indicates *p* < .05, ** indicates *p* < 0.005, *** indicates *p* < 0.0005 and **** indicates p < 0.00005. (A-D) is representative of 4 independent experiments; (E) is representative of 2 independent experiments.

It has been reported that with age the proportion of myeloid cells and cytokines in the blood is increased. We quantitated circulating leukocyte populations in old (18–22 mo) mice and found that, consistent with previously published data[47,48], there was a decrease in the percentage of T cells and an increase in the number of myeloid cells when compared with young (10–14 wk) mice (Fig 1A & S1A Fig). Analysis of monocyte subsets indicated that the absolute number of both Ly6C^high^ and Ly6C^low^ monocytes was increased with age (Fig 1A). An increase in Ly6C^high^ monocyte frequency within the blood of old mice was paralleled by a similar increase in the bone marrow (Fig 1B), suggesting that increased myelopoiesis within the bone marrow may precede increased numbers of these cells in the blood. Consistent with this, we also found that the number of M-CSF responsive cells (myeloid precursors and monocytes capable of differentiating into bona fide macrophages *ex vivo*) in the bone marrow was significantly increased with age (S1C Fig).

The C-C chemokine receptor type 2 (CCR2) is expressed at high levels on Ly6C^high^ monocytes and is essential for their entry into the blood in response to the production of CCL2[49]. Since CCR2 is required for monocytes, and especially Ly6C^high^ monocytes, to leave the bone marrow and enter the blood, we hypothesized that enhanced CCR2 expression on Ly6C^high^ monocytes could prompt their premature emigration from the bone marrow and could explain the increased number of Ly6C^high^monocytes seen with age. CCR2 expression was measured on Ly6C^high^ monocytes in the blood and bone marrow of old mice and found to be dramatically increased (Fig 1C). Consistent with previous research[1], CCR2 expression was more pronounced on Ly6C^high^ monocytes (S1E Fig). As Ly6C^high^ monocytes represent an intermediate stage in monocyte-to-macrophage differentiation, we investigated their maturity using the monocyte/macrophage maturity marker, F4/80. Interestingly, we found that there was an inverse relationship between CCR2 expression and F4/80 expression on Ly6C^high^ monocytes in the blood of old mice. With age, these cells showed significantly decreased levels of F4/80 (Fig 1D), suggesting that their increased CCR2 expression may prompt these cells to enter the circulation in an immature state. When CCR2 expression was measured on myeloid precursors undergoing M-CSF-stimulated differentiation into macrophages, increased CCR2 expression occurred during an intermediate stage of differentiation (day 5) on cells from old mice (S1D Fig).

To determine whether increased CCR2 expression was sufficient to increase Ly6C^high^ monocyte egress, we intraperitoneally injected young and old mice with 100 nM of CCL2 and measured Ly6C^high^ monocyte recruitment after 4 hours. We found that despite administering an equivalent dose of CCL2, Ly6C^high^ monocyte recruitment to the peritoneum was increased ~5-fold in old mice relative to young mice (Fig 1E). A less dramatic increase in Ly6C^low^ monocytes was also observed (Fig 1E), consistent with previous studies.

### Monocytes are potent producers of pro-inflammatory cytokines with age

**Fig 2:**
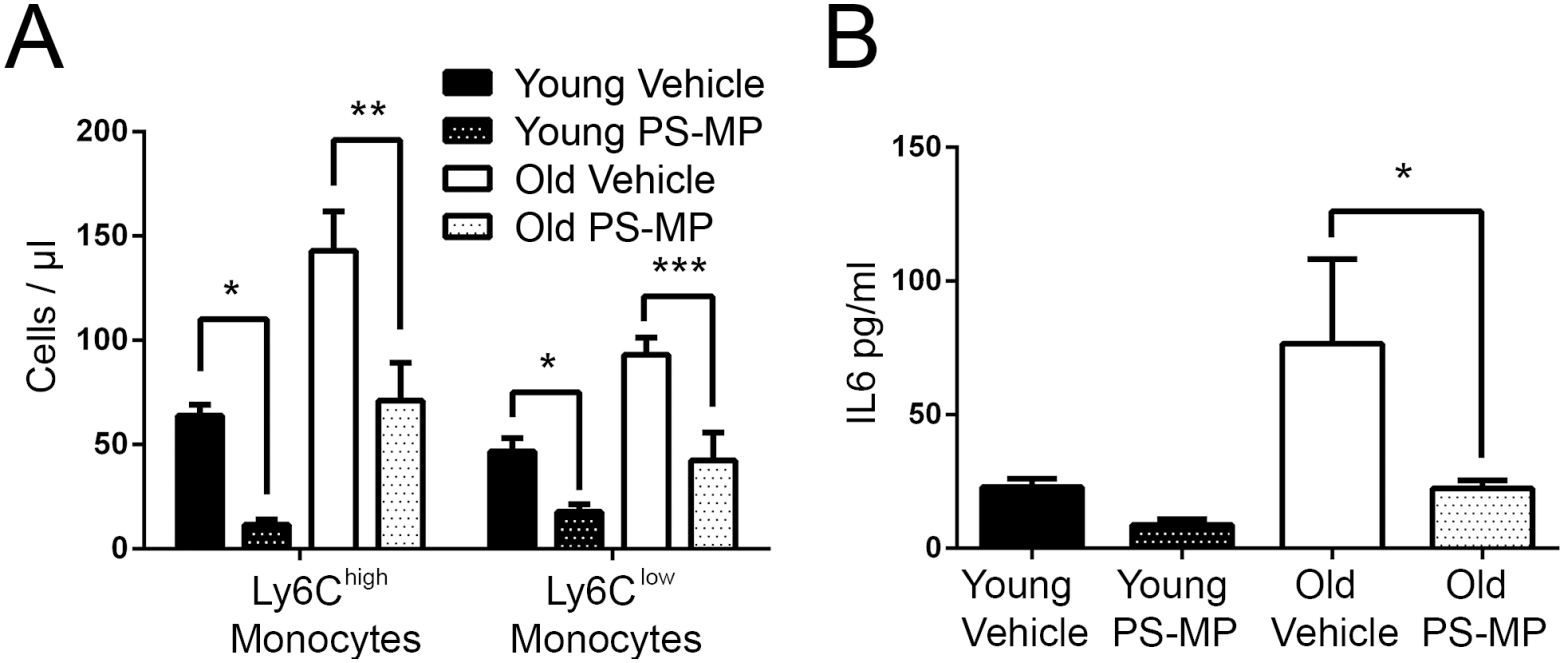
Ly6C^high^ monocytes contribute to elevated levels of serum IL6 and TNF in aged mice. Young and old mice were injected with 500 nm negatively-charged polystyrene microparticles (PS-MPs) previously shown to reduce numbers of circulating Ly6C^high^ monocytes. Circulating monocyte populations (A) and IL6 levels in whole blood (B) were quantitated after 24 hours. Statistical significance was determined by two-tailed Mann-Whitney-Wilcoxon test. * indicates *p* < .05, ** indicates *p* < 0.005, *** indicates *p* < 0.0005 and **** indicates p < 0.00005. (A-B) is representative of ± SEM of 5 mice from 2 independent experiments.

**Fig 3:**
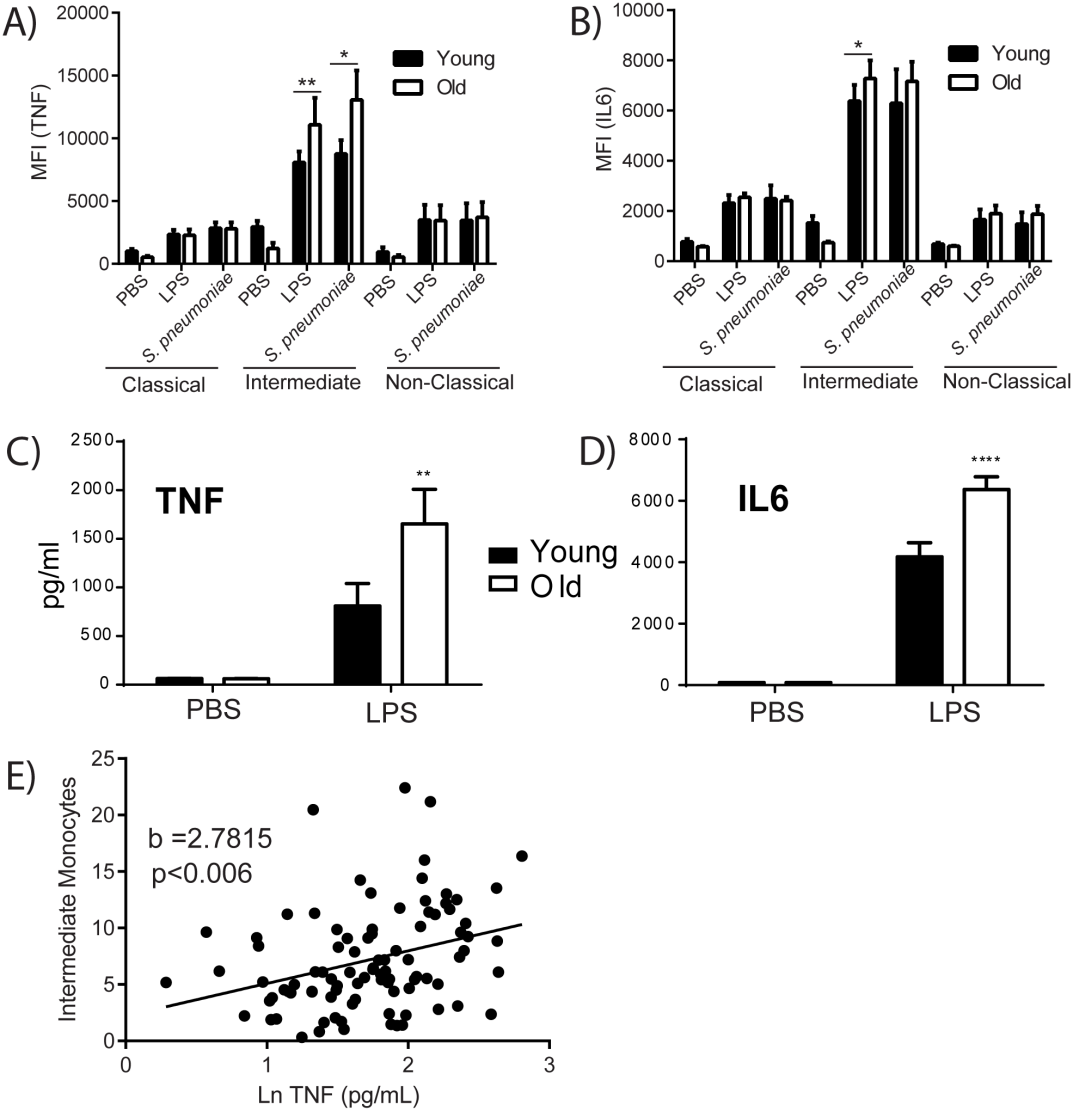
Human CD14^++^CD16^+^HLA-DR^+^ (intermediate) monocytes produce more inflammatory cytokines with age. Intracellular production of TNF (A) and IL-6 (B) in classical (CD14^++^), intermediate (CD14^++^CD16^+^) and non-classical (CD14^+^CD16^+^) monocytes from young and elderly donors in response to LPS (50 ng/ml) and *S*. *pneumoniae (5 x10* ^*6*^ *CFU)*. C) The secretion of TNF and D) IL-6 for isolated CD14+ monocytes in response to LPS for young and older donors. E) The frequency of intermediate monocytes were found to have a significant, positive correlation with the levels of serum TNF (β = 2.78, p<0.006). (A-D) is representative of ± SEM of n = 7 young donors (26–52 yrs) and n = 6 older donors (63–70 yrs) *indicates p<0.05, and ** indicates p< 0.05. Intermediate monocyte (CD14++CD16+HLA-DR+) count (cells per microlitre of whole blood) increases relative to serum levels of TNF in older donors (n = 94, 61-100yrs).

Since we found that there was an expansion of monocytes with age and these cells are known to be potent producers of pro-inflammatory cytokines, we postulated that they might contribute significantly to age-associated inflammation. To determine whether the increased numbers of monocytes with age contributed to age-associated increases in IL6 production, we targeted this cell population using carboxylated polystyrene microparticles (PS-MPs), which have been shown by others to lead to a reduction of primarily Ly6C^high^ monocytes in the blood[50]. We found that when circulating monocytes were decreased in old mice (Fig 2A), this reduced circulating levels of IL6 (Fig 2B). In humans, CD14^++^CD16^+^HLA-DR^+^/intermediate monocytes are the biggest producers of inflammatory cytokines under a variety of stimulation conditions [3]. Intracellular cytokine staining reveals that of the three human monocyte populations (classical, intermediate, non-classical) intermediate monocytes are the major producers of TNF (Fig 3A) and IL6 (Fig 3B) after stimulation with LPS or *S*. *pneumoniae* and older donors (63–70 yrs) produce more cytokines than younger donors (26–52 yrs). Additionally, CD14^+^ monocytes isolated from PBMCs from older donors produced more TNF (Fig 3C) and IL6(Fig 3D) in response to LPS than did younger donors. As in mice, the numbers of intermediate monocytes may be influenced by levels of age-associated inflammation since the frequency of intermediate monocytes, are positively correlated with plasma TNF (Fig 3E) as has been shown to occur in other chronic inflammatory conditions [51]. A weaker correlation (p<0.02) was observed between TNF levels and the numerically dominant classical monocytes and no correlation was found between non-classical monocytes and TNF (p = 0.2).

### The age-associated increase in circulating pro-inflammatory monocytes is regulated by TNF

**Fig 4:**
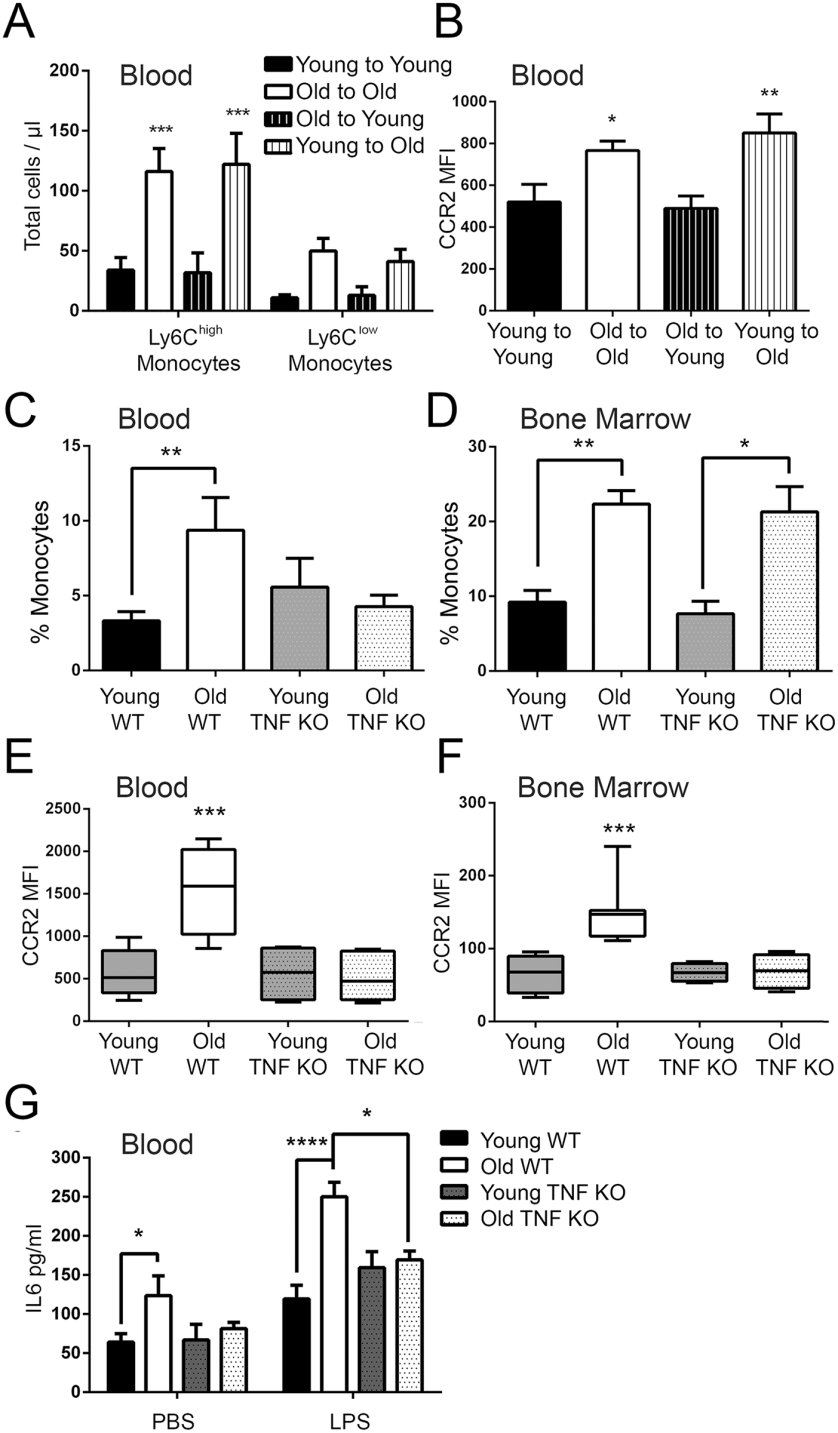
TNF drives increases in circulating Ly6C^high^ monocytes. (A) Total numbers of Ly6C^high^ and Ly6C^low^ monocytes in the blood of heterochronic bone marrow chimeric mice. Old recipient mice which receive young donor marrow have increased numbers of circulating Ly6C^high^ and Ly6C^low^ monocytes which are comparable to old recipient mice that receive old donor marrow. Young recipient mice that receive old donor marrow do not have an increase in Ly6C^high^ and Ly6C^low^ monocytes. The data represent the mean (± SEM) of 5 mice. (B) CCR2 expression on circulating monocytes is elevated when the recipient mouse is old, indicating that the bone marrow microenvironment drives changes in CCR2 expression (CCR2 MFI± SEM; *n* = 5). (C-D) The percent Ly6C^high^ monocytes as a proportion of CD45^+^ cells in the (C) blood or (D) bone marrow of young and old WT and TNF KO mice was quantitated (± SEM; *n* = 4–6). (E-F) Expression of CCR2 on Ly6C^high^ monocytes in the (E) blood or (F) bone marrow of young and old WT and TNF KO mice (*n* = 4–8) demonstrate that the presence of TNF drives CCR2 expression with age. (G) IL6 production in whole blood from young and old TNF KO mice stimulated with 100 ng/ml of LPS or a vehicle control for 24 hours was quantitated by ELISA (± SEM; *n* = 5). Statistical significance was determined by two-tailed Mann-Whitney-Wilcoxon test, one-way or two-way ANOVA with Fisher's LSD post-test where appropriate. * indicates *p* < .05, ** indicates *p* < 0.005, *** indicates *p* < 0.0005 and **** indicates *p* < 0.00005. (A-B) is representative of 2 independent experiments; (C-G) is representative of 3 independent experiments.

To determine whether age-related changes in Ly6C^high^ monocyte numbers, phenotype and inflammatory capacity were caused by changes in the aging bone marrow microenvironment or due to intrinsic changes in the myeloid precursors themselves, we created heterochronic bone marrow chimeras. When young bone marrow was transferred to old recipient mice the number of Ly6C^high^ and Ly6C^low^ monocytes was increased to levels comparable to old mice (Fig 1A) or old recipient mice who had received old donor marrow (Fig 4A). In contrast, young recipient mice that had received old donor marrow had Ly6C^high^ and Ly6C^low^ monocyte numbers comparable to young mice (Fig 1A) or to young recipient mice that had received young donor bone marrow (Fig 4A). In addition, the increase in CCR2 expression observed on circulating monocytes from old mice (Fig 1C) was also observed in circulating monocytes from old recipient mice who had received young donor marrow but not on young recipient mice who received old donor marrow(Fig 4B). These data demonstrate that increases of Ly6C^+^ monocytes and increased CCR2 expression occur in a manner entirely dependent on the bone marrow microenvironment.

Since TNF is one of the central cytokines associated with inflamm-aging, we investigated whether TNF was sufficient to drive expansion of the Ly6C^high^ monocytes. We aged TNF knockout (KO) mice (18–22 mo) and quantified Ly6C^high^ monocytes in their blood. We found that, unlike their WT counterparts, old TNF KO mice did not have higher numbers of circulating Ly6C^high^ monocytes (Fig 4C), but did have an increase in bone-marrow Ly6C^high^ monocytes compared to their young counterparts (Fig 4D). Surface expression of CCR2 on Ly6C^high^ monocytes in both the blood (Fig 4E) and the bone marrow (Fig 4F) of old TNF KO mice was comparable to the levels seen in young mice. Similarly there were no changes in Ly6Clow monocytes in aged TNF KO mice (S1D Fig).

These data suggest that increased production of Ly6C^high^ monocytes in the bone marrow occur independent of TNF, but that increases in CCR2 expression on these cells in the bone marrow, and their subsequent mobilization to the blood is TNF-dependent. Consistent with our observation that Ly6C^+^monocytes contribute to elevated levels of circulating cytokines with age (Fig 2), old WT mice produced more IL6 than young mice following 24 hour stimulation of whole blood with either PBS or LPS (Fig 4G). In comparison, old TNF KO mice, which did not have an increase of Ly6C^+^monocytes in the blood did not have an age-associated increase in IL6 in whole blood in response to PBS or LPS (Fig 4G).

### Blockade of TNF reverses age-associated increases in Ly6C^high^ monocytes and inflammation

**Fig 5:**
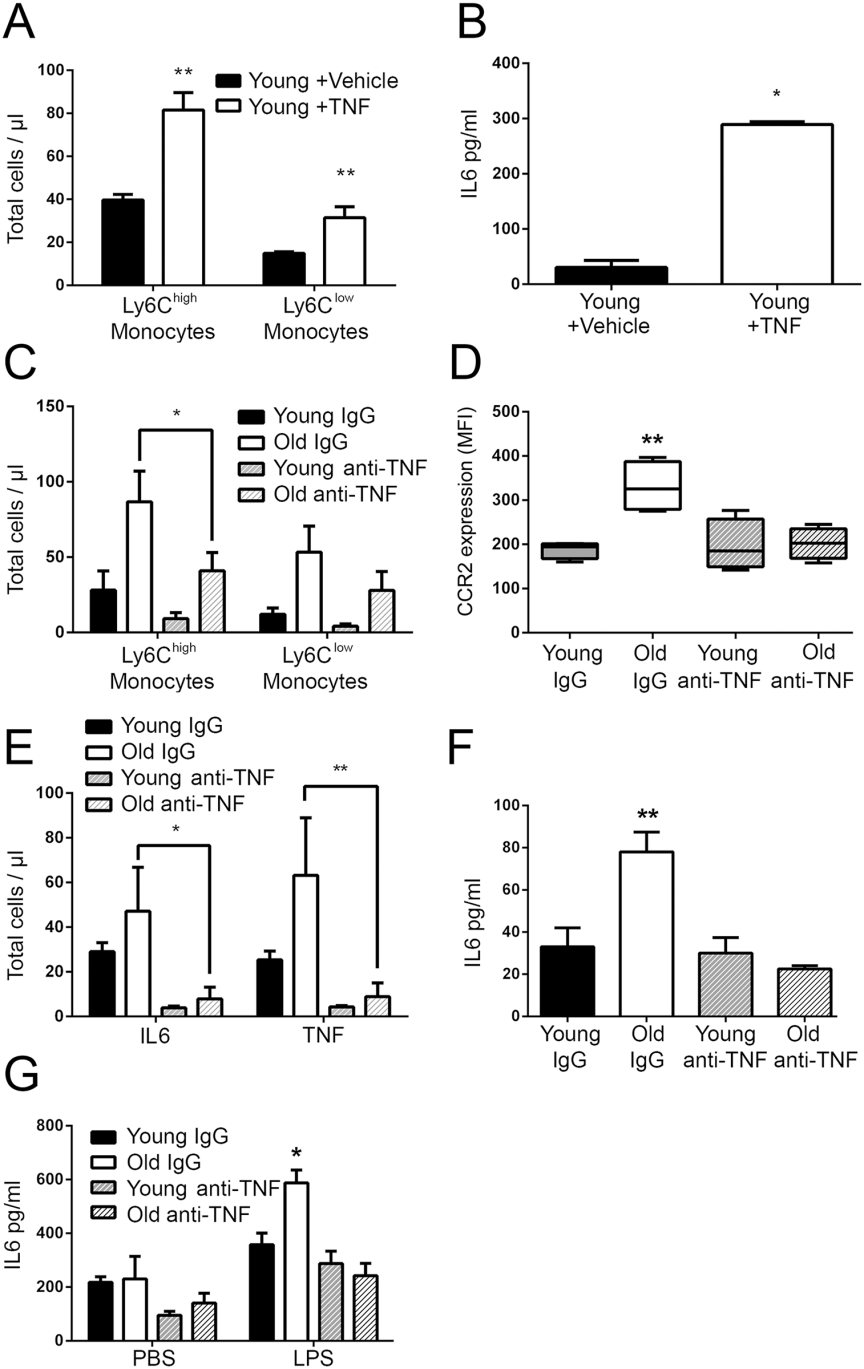
Anti-TNF therapy can reverse the age-associated increase in circulating Ly6C^high^ monocytes. (A-B) Young mice were give 200 ng/ml of TNF intraperitoneally every other day for 3 weeks. Numbers of circulating Ly6C^high^ and Ly6C^low^ monocytes (A) and serum IL6 (B) were quantitated. The data represent the mean (± SEM) of 5 mice. (C) Young and old WT mice were treated for 3 weeks with a neutralizing TNF antibody or IgG control and total numbers of circulating Ly6C^high^ monocytes were quantitated by flow cytometry. The data represent the mean (± SEM) of 4 mice. (D) The mean CCR2 expression on circulating Ly6C^high^ monocytes in young and old mice treated with either anti-TNF or IgG was quantitated and found to be reduced with anti-TNF treatment (*n* = 4). (E) Intracellular staining of IL6 and TNF on blood monocytes after a 4 hour stimulation with LPS from young and old WT mice treated with either anti-TNF or IgG demonstrates that the number of monocytes that stain positive for IL6 or TNF are decreased with anti-TNF therapy(± SEM; *n* = of 4). (F) Serum IL6 is reduced in old mice treated with anti-TNF but not the IgG control. (G) IL6 production in whole blood following stimulation with LPS or a vehicle control after 24 hours from young and old WT mice given either anti-TNF or IgG (± SEM; *n* = 4). Statistical significance was determined by two-tailed Mann-Whitney-Wilcoxon test, one-way or two-way ANOVA with Fisher's LSD post-test where appropriate. * indicates *p* < .05, ** indicates *p* < 0.005, *** indicates *p* < 0.0005 and **** indicates *p* < 0.00005. (A-G) are representative of 1 experiment with n = 4 mice.

We investigated whether it was chronic or acute exposure to TNF that mediated age-related increases in serum IL6 and changes in monocyte phenotype and function. We first sought to determine whether increases in circulating Ly6C^+^ monocytes were inducible after administration of TNF. TNF (5ng/g) was administered intraperitoneally for 3 weeks, a time point chosen because it would allow for multiple cycles of monopoiesis and complete turnover of pre-formed monocytes [52]. Young mice showed a large increase in Ly6C^high^ monocytes in the blood and a less dramatic increase of Ly6C^low^ monocytes (Fig 5A). This was accompanied by a significant increase in serum IL6 in TNF-treated, but not vehicle control mice (Fig 5B). We next asked whether blocking TNF could reduce numbers of Ly6C^+^ monocytes in old animals. Young and old WT mice were administered Adalimumab (HUMIRA), a human monoclonal antibody specific for TNF, or an IgG isotype control at a dose of 50 ng/g for a period of three weeks via intraperitoneal injection. Anti-TNF therapy reduced the levels of plasma TNF from an average of 1.5 pg/ml to below the level of detection (LOD = 0.25pg/ml) in old mice and decreased the number of circulating Ly6C^high^ but not Ly6C^low^ monocytes in the blood to levels similar to young mice (Fig 5C). Anti-TNF therapy also reduced CCR2 expression on Ly6C^high^ monocytes in the blood of old mice to levels that are equivalent to those seen in young mice (Fig 5D) and reduced the percentage of monocytes that stained positive for IL6 or TNF by ICS after LPS stimulation (Fig 5E). Anti-TNF treatment reduces IL6 levels in the circulation of old mice (Fig 5F) and when blood from young and old mice treated with anti-TNF or IgG controls was stimulated with LPS, IL6 levels were lower in old mice treated with anti-TNF compared to those that were treated with IgG(Fig 5G).

### Circulating and recruited Ly6C^high^ monocytes are increased with age during *S*. *pneumoniae* colonization

**Fig 6:**
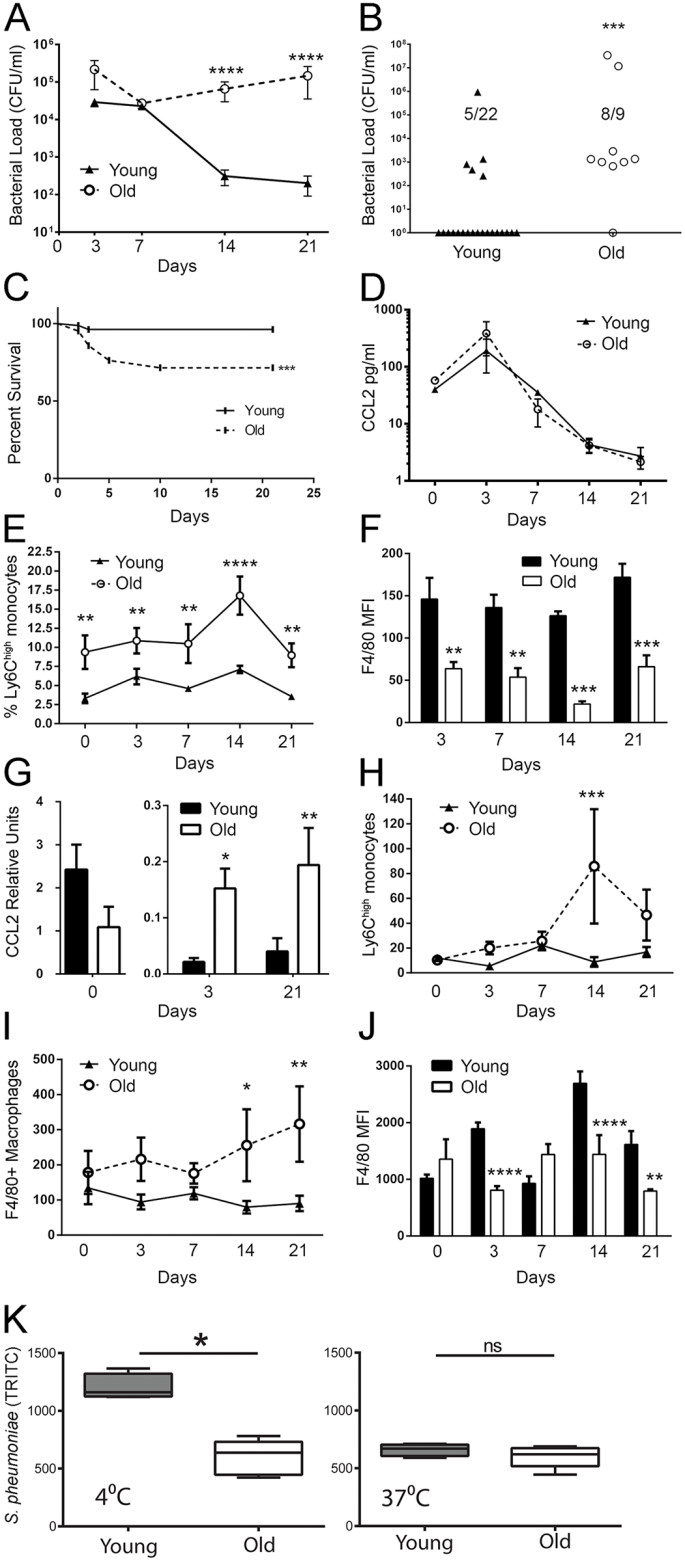
Old mice have increased numbers of circulating and recruited Ly6C^high^ monocytes during the course of *S*. *pneumoniae* colonization. (A) Colony forming units (CFUs) in nasal lavages from young and old WT mice were quantified on days 3, 7, 14 and 21 following intranasal colonization with *S*. *pneumoniae* (± SEM; *n* = 5–22). (B) CFUs of *S*. *pneumoniae* in the lungs at day 3 following intranasal colonization (± SEM; *n* = 9–22). (C) Survival of young and old mice after intranasal *S*. *pneumoniae* colonization (± SEM; *n* = 12). (D) Total serum CCL2 in young and old mice following intranasal *S*. *pneumoniae* colonization was measured by a high sensitivity ELISA. The data represent the mean (± SEM) of 3 mice per time point. (E) Ly6C^high^ monocytes as a percent of CD45^+^ cells in the blood of young and old WT mice during nasopharyngeal *S*. *pneumoniae* colonization (± SEM; *n* = 5–8) was measured by flow cytometry. (F) Mean expression of F4/80 on Ly6C^high^ monocytes in the blood of old mice during *S*. *pneumoniae* colonization is decreased as compared to young mice. (G) Levels of CCL2 transcript in the nasopharynx during the course of *S*. *pneumoniae* colonization were measured by quantitative PCR. (± SEM; *n* = 3). (H-I) Total numbers of (H) Ly6C^high^ monocytes and (I) macrophages detected by flow cytometry in the nasopharnyx of young and old mice during *S*. *pneumoniae* colonization (± SEM; *n* = 3–8). (J) Mean F4/80 expression on nasopharyngeal macrophages is lower in old mice during *S*. *pneumoniae* colonization (± SEM; *n* = 3–8). Statistical significance was determined by two-tailed Mann-Whitney-Wilcoxon test, one-way or two-way ANOVA with Fisher's LSD post-test. (K) Circulating blood monocytes from old mice bind fewer TRITC-labelled *S*. *pneumoniae* (4°C) but there is no difference in internalization of the bacteria (37°C). Survival in (C) was determined by the Mantel-Cox Log-rank test. * indicates *p* < .05, ** indicates *p* < 0.005, *** indicates *p* < 0.0005 and **** indicates p < 0.00005. (A-J) is representative of 3 independent experiments.

In order to determine if age-related changes in Ly6C^high^ monocyte numbers or maturity might play a role in defective anti-bacterial immunity with age, we investigated the trafficking of these cells following nasopharyngeal colonization of young and old mice with the bacterial pathogen, *S*. *pneumoniae*. We selected this pathogen specifically because of the high burden of disease caused by *S*. *pneumoniae* in elderly individuals and because it has been previously demonstrated that its clearance from the nasopharynx is largely dependent on recruited monocytes/macrophages[53,54]. Following intranasal delivery of *S*. *pneumoniae*, we found that old mice had defects in clearance of the colonization. By Day 21 most of the young mice had cleared the bacteria, while old mice still harbored high bacterial loads (Fig 6A). Old mice were also more susceptible to bacterial invasion to the lungs at day 3 (Fig 6B) and mortality throughout the course of colonization (Fig 6C). Although serum production of CCL2 in old mice was comparable to that of young mice (Fig 6D), old mice had increased Ly6C^high^ but not Ly6C^low^ monocyte numbers in the circulation during colonization (days 3, 7, 14, 21) (Fig 6E).

We next investigated whether the monocytes/macrophages recruited in the context of age had maturity defects (as measured by F4/80 expression). In old mice, circulating Ly6C^high^ monocytes had decreased expression of F4/80 during colonization (Fig 6F), suggesting that the decreased F4/80 expression seen in the bone marrow during the steady state (Fig 1D) perpetuates following their egress during infectious challenge. Despite their inability to control bacterial loads in the nasopharynx, old mice also had a significant increase in the expression of CCL2 in the nasopharynx during colonization (Fig 6G), and had higher numbers of recruited Ly6C^high^ monocytes (Fig 6H) and macrophages (Fig 6I) to the nasopharynx compared to young mice. Although resident macrophages from young and old mice present in the nasopharynx during the steady state expressed equal levels of F4/80, monocytes/macrophages recruited to the nasopharynx during *S*. *pneumoniae* colonization showed decreased expression F4/80(Fig 6J), similar to that seen in their counterparts in the blood(Fig 6F). In order to determine whether bacterial binding and internalization was different between monocytes derived from young and old mice we compared bacterial binding (measured at 4°C) and internalization/killing (measured at 37°C). Although there was a significant decrease in bacterial binding between young and old mice, this did not appear to affect internalization or bacterial killing (Fig 6K).

### Ly6C^+^ monocytes impair clearance of *S*. *pneumoniae* with age

**Fig 7:**
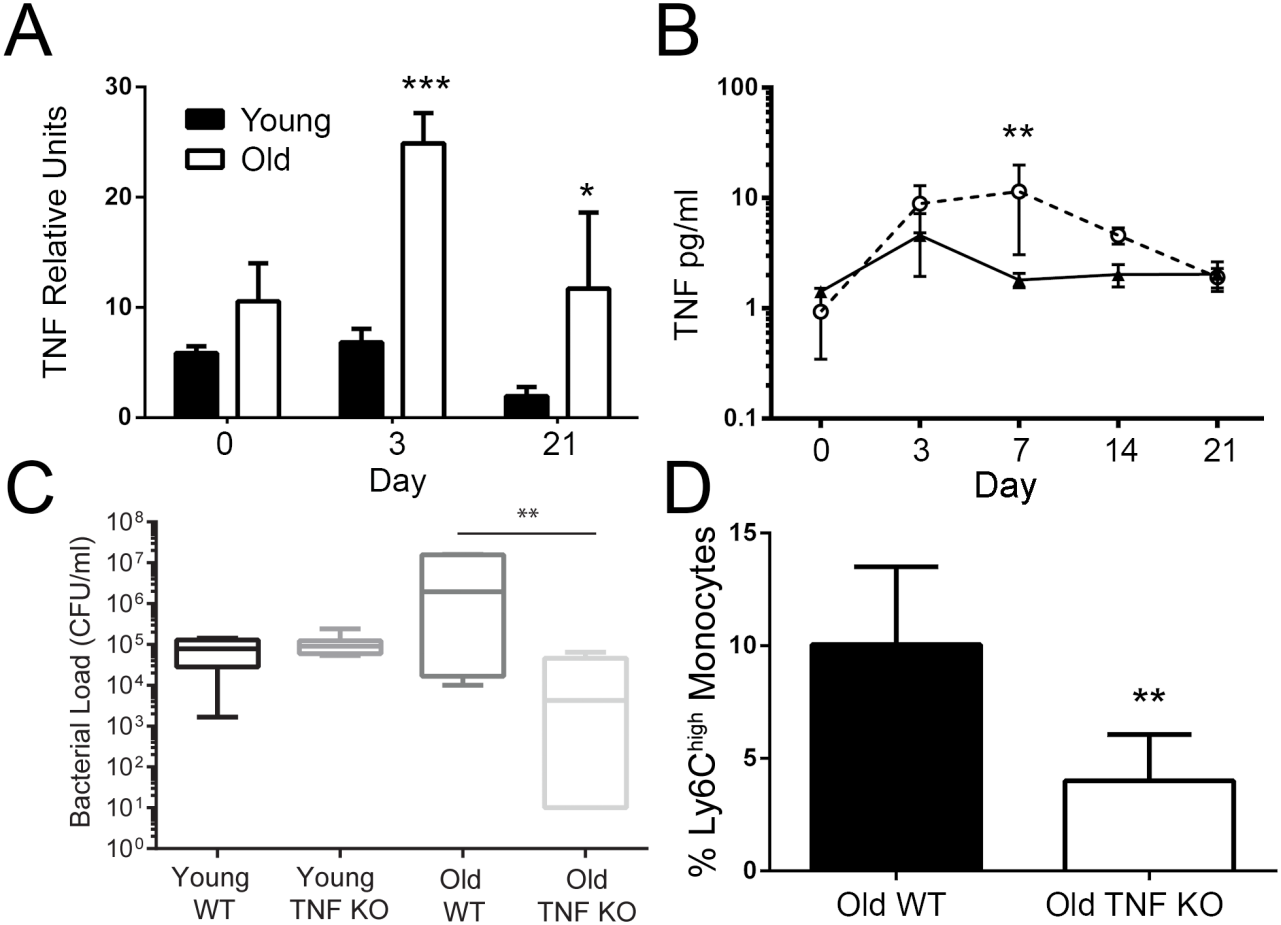
Reducing TNF-regulated recruitment of Ly6C^high^ monocytes during *S*. *pneumoniae* colonization in old mice reduced nasopharyngeal bacterial loads. (A-B) TNF in the (A) nasopharnyx and (B) serum of young and old mice during *S*. *pneumoniae* colonization as measured by qPCR and ELISA, respectively (± SEM; *n* = 3–5). (C) CFUs in nasal lavages of old WT and old TNF mice on day 4 after colonization with *S*. *pneumoniae* (± SEM; *n* = 6–8, one independent experiment of two shown). (D) Ly6C^high^ monocytes as a percent of circulating CD45+ cells in old WT and TNF KO mice on day 4 of *S*. *pneumoniae* colonization (± SEM; *n* = 3–4, one independent experiment of two shown). Statistical significance was determined by two-tailed Mann-Whitney-Wilcoxon test, one-way ANOVA or two-way ANOVA with Fisher's LSD post-test where appropriate. * indicates *p* < .05, ** indicates *p* < 0.005, *** indicates *p* < 0.0005 and **** indicates p < 0.00005.

Although trafficking of blood monocytes was not impaired with age, old mice nonetheless displayed impaired clearance of *S*. *pneumoniae*. To explain this, we hypothesized that high levels of recruited but developmentally immature Ly6C^high^ monocytes could, in fact, have negative consequences for clearance. Interestingly, TNF, which we showed caused increased numbers of Ly6C^high^ monocytes in the blood (Fig 4A), was increased with age during *S*. *pneumoniae* colonization in the nasopharynx (Fig 7A) and blood (Fig 7B). We next compared nasopharyngeal bacterial loads in WT and TNF KO mice, to determine whether TNF production affected bacterial clearance. Although TNF had no effect on clearance of colonization in young mice we found that old TNF KOs had significantly fewer CFUs in the nasopharnyx compared to their old WT counterparts at day 3 (Fig 7C). Old TNF KO mice also had decreased recruitment of Ly6C^high^ monocytes in the blood (Fig 7D), confirming that TNF can regulate mobilization of these cells during infection as well as in the steady state.

**Fig 8:**
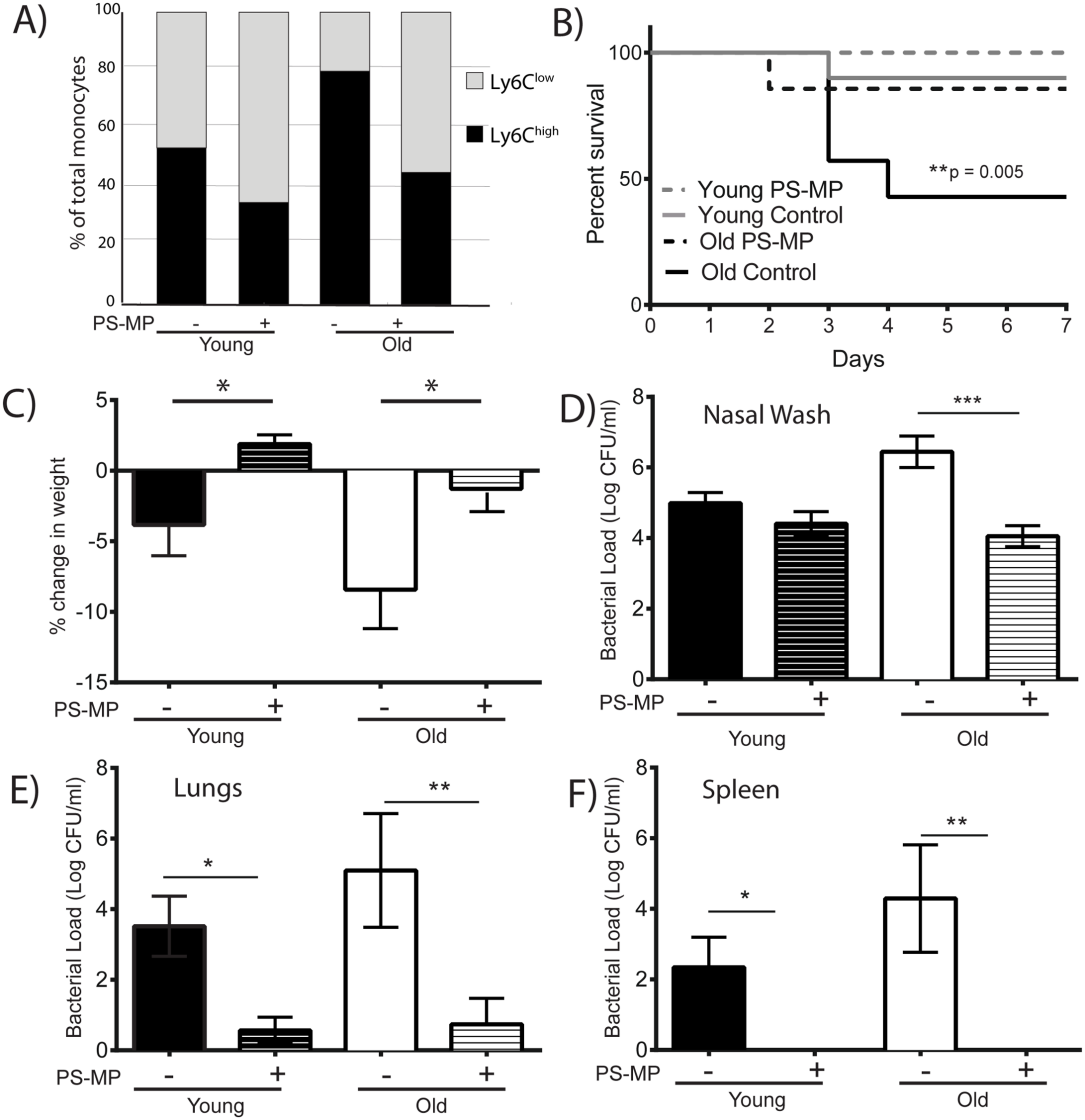
Depletion of inflammatory monocytes improves outcome to S. pneumoniae infection in old mice. Mice (n = 7-10/group) were injected with PS-MP day -1, 0, +1, +3 and +5 during colonization *with S*. *pneumoniae*. A) The percentage of Ly6C^high^ monocytes was significantly reduced in old mice treated with PS-MP (see S3 Fig). B) Survival was significantly improved in old mice treated with PS-MP (p = 0.005, Mantel-Cox log-rank test). C) Both young and old mice treated with PS-MP lost less weight than their control counterparts (*,p<0.05, one-way ANOVA with uncorrected Fisher's LSD). Levels of *S*. *pneumoniae* in the D) nasal wash, E) lungs and F) spleen were lower in old mice treated with PS-MP. Fewer young mice had bacteria in their lungs and spleens when they were treated with PS-MP. (*,p<0.05, **,p<0.005 one-way ANOVA with uncorrected Fisher's LSD). CFU count for mice that reached endpoint before day 7 are not included.

To determine whether the decreased recruitment of Ly6C^high^ monocytes we observed was responsible for improved bacterial clearance in old TNF KO mice, we preferentially targeted this cell population using negatively-charged polystyrene microparticles (PS-MPs) (Fig 8A). We observed that there were also decreases in monocytes in the lungs, but not neutrophils with this treatment (S2 Fig). Old mice were given PS-MPs on day prior to and every 3 days during the course of *S*. *pneumoniae* colonization and bacterial loads were measured at day 7. PS-MP-treated old mice had increased survival (Fig 8B), less weight loss (Fig 8C)and lower bacterial loads in the nasopharynx (Fig 8D), lungs (Fig 8E) and spleen (Fig 8F) compared to old control mice. Similar results were observed with Gr-1 antibody, which reduces numbers of monocytes and neutrophils. These data confirm that increased trafficking of this cell type during *S*. *pneumoniae* colonization impairs host defense.

## Discussion

Epidemiological data strongly suggests that there is a reciprocal link between pneumonia and age-associated inflammation. Older adults who have higher than age-average levels of the cytokines TNF and IL6 in their circulation have a much higher risk of acquiring pneumonia than their peers who have lower than age-average levels[55]. Although a robust inflammatory response is generally thought to be protective against infection, in the elderly, high levels of circulating inflammatory cytokines during pneumonia are associated with more severe disease and higher mortality[56,57]. Similarly, having a chronic inflammatory disease such as dementia, diabetes, or cardiovascular disease is strongly associated with susceptibility to acquiring pneumonia [58,59,60]. Conversely, having a pneumonia in mid- to late-life can often exacerbate or accelerate sub-clinical or existing chronic inflammatory conditions and can be the harbinger of declining health and decreased quality of life[58,59]. Although descriptions of this reciprocal relationship between chronic, age-associated inflammation and pneumonia, especially that caused by *S*. *pneumoniae*, are strong, the mechanistic explanations are weak. Herein we demonstrate that monocytes, both contribute to age-associated inflammation and are impaired by chronic exposure to the inflammatory cytokine TNF, and this ultimately impairs their anti-pneumococcal function.

Our data using aged TNF KO mice or anti-TNF therapy indicate that the increased levels of TNF that occur with age impair monocyte development and ultimately anti-bacterial immunity. Although macrophages have previously been shown to promote inflamm-aging[61], our data suggest that this may begin earlier in myelopoesis since monocytes produce more inflammatory cytokines such as TNF and IL6 with age and ablation of monocytes reduces levels of serum cytokines. The increase in circulating monocytes did not occur in old TNF KO mice. Furthermore, by treating young WT mice with a low-dose regime of TNF delivered intraperitoneally, we found that Ly6C^+^ monocytes were increased in the blood in a manner similar to old mice, demonstrating that TNF is sufficient to increase numbers of circulating Ly6C^+^ monocytes. Monocytes appear to be both highly responsive to increased levels of TNF but also seem to be a major source of age-associated TNF.

Our observational studies in humans imply that the numbers of intermediate (CD14^++^CD16^-^) monocytes, which we have previously shown express higher levels of CCR2 with age [62], correlate with increased levels of TNF and contribute to hyper-inflammatory responses to bacterial infection. Studies in patients on anti-TNF therapy for rheumatoid arthritis validate our observations that TNF drives increases in inflammatory monocytes. In these patients anti-TNF therapy decreases the levels of circulating CD14^++^CD16^-^ monocytes in the blood and synovial fluid as well as decreases CCR2 expression on peripheral blood mononuclear cells and thus is consistent with our data demonstrating that TNF-mediated changes in CCR2 expression are sufficient to alter the numbers of Ly6C^high^ monocytes in the circulation [63,64]. Interestingly, decreases in CD14^++^CD16^-^ monocytes correlate with a positive prognostic response for patients, but whether this is because they contribute directly to disease progression or the inflammatory tone of rheumatoid arthritis is not known [63].

Increases in Ly6C^high^ monocytes are associated with defects in maturity. Interestingly, our chimera data demonstrate that phenotypic changes in monocytes (i.e. CCR2 and F4/80 expression) were not due to intrinsic defects in myeloid precursors but rather the influence of the bone marrow microenvironment, and, since these changes did not occur in TNF KO mice, TNF produced in the context of the microenvironment. Although F4/80 levels were equivalent on blood monocytes during the steady state, they were lower on Ly6C^high^ monocytes/differentiating macrophages recruited during nasopharyngeal *S*. *pneumoniae* colonization in old mice. These changes had functional significance; despite robust Ly6C^high^ monocyte recruitment and TNF production in old mice, bacterial clearance was significantly impaired. In fact, our data suggest that TNF is detrimental to clearance of *S*. *pneumoniae* from the nasopharynx with age, as old TNF KO mice had lower bacterial loads compared to their WT counterparts. Although TNF is often thought of as a key anti-bacterial cytokine, mouse studies have demonstrated that TNF is required for control for *S*. *pneumoniae* bacteremia but not for survival in lung infection[65]. In our study, old TNF KO mice recruited fewer circulating Ly6C^high^ monocytes during *S*. *pneumoniae* colonization compared to old WT mice and counter-intuitively, this appeared to be protective against infection as when we depleted circulating Ly6C^high^ monocytes using carboxylated polystyrene microparticles colonization, bacterial loads in the nasopharynx decreased. These data are consistent with the clinical observation that rheumatoid arthritis patients (who have high levels of circulating TNF) are at increased risk of pneumonia but that there is no increase in risk of pneumonia for patients on anti-TNF therapy [66]. Whether pneumonia risk is *decreased* with anti-TNF therapy is not known; however, patients on anti-TNF therapy do live slightly longer than their untreated counterparts, despite an increased risk in re-activation of chronic infections[67,68].

These observations have important therapeutic significance, since the belief that host responses to bacteria are impaired with age due to poor innate cell recruitment has been the foundation of two large clinical trials testing the use of cytokines (G-CSF) to mobilize myeloid cells as an adjunct to antibiotics and one clinical trial testing GM-CSF as an adjuvant for pneumococcal vaccination. Although mouse models (tested in young mice) showed promise, these strategies all failed when tested in populations where the median ages were 59, 61 and 68, respectively [reviewed in[69] and[70]]. Our data suggests that use of G-CSF, GM-CSF or other myeloid chemoattractant-based therapies in older adults would enhance recruitment of a population that is fundamentally immature and predisposed towards TNF and IL6 production that provides no functional benefit to the host for clearance and may even exacerbate infection.

In summary, our data suggest that monocytes are both contributors to age-associated inflammation and have altered anti-pneumococcal function as a result of age-associated inflammation. Lowering levels of TNF may be an effective strategy in improving host defence against *S*. *pneumoniae* in older adults. In fact, it has been shown that immunosuppressive steroid use in combination with antibiotics reduces pneumonia mortality in the elderly[71,72,73,74], although uptake for this therapy has been limited. Although it may be counterintuitive to limit inflammatory responses during a bacterial infection, the clinical observations and our animal model indicates that anti-bacterial strategies need to be tailored to the age of the host.

## Materials and Methods

### Ethics statement

All experiments were performed in accordance with Institutional Animal Utilization protocols approved by McMaster University’s Animal Research Ethics Board (#13-05-13 and #13-05-14) as per the recommendations of the Canadian Council for Animal Care.

Participants or Power of Attorney for participants were approached to determine interest in the study. Informed written consent was obtained from the participant or their legally authorized representative approved by the Hamilton Integrated Research Ethics Board (#09–450).

### Animals

Female C57BL/6J mice were purchased from Jackson Laboratories and aged in house. Colonization was performed as previously described[75]. To protect from age-related obesity aging mice are fed with a low protein diet Teklad Irradiated Global 14% protein Maintenance Diet and provided with an exercise wheel, as were young controls. The average weight of a young mouse is this study is 20g+/-1g and the old mice are on average, 27g+/-2.5g. TNF knockout mice (KO) mice (C57BL/6J background) were bred in the barrier unit at the McMaster University Central Animal Facility (Hamilton, ON, Canada) as previously described[76]. All mice were housed in specific pathogen-free conditions. Continual monitoring of the health status of mice was performed.

### Human monocytes

Monocyte frequency was measured in whole blood according to staining procedures described in [62]. Briefly, intermediate monocytes were positive for the expression of HLA-DR and CD16, stained brightly for CD14, and were negative for lymphoid and neutrophil markers (CD2, CD3, CD15, CD19, CD56, and NKp46). They are presented as cells per microlitre of whole blood, which was measured using CountBright Absolute Counting Beads (Life Technologies, CA, USA). Serum TNF was measured in elderly donors (61–100 yrs) using the Milliplex High Sensitivity ELISA kit (Millipore, ON, CA).

For intracellular cytokine staining, described in [62], the production of TNF and IL-6 was measured in classical (CD14++), intermediate (CD14++CD16+) and non-classical (CD14+CD16+) monocytes after a 6 hour incubation period in the presence of 50 ng/ml LPS and 5 x 10^6^ CFU of heat-killed *S*. *pneumoniae*. For cytokine secretion, CD14+ monocytes were isolated from PBMCs of young(26–52 yrs) and older (63–70 yrs)by positive selection procedure (Stemcell, BC, CAN) and stimulated for 22 hours in the presence of 50 ng/ml LPS. TNF and IL-6 were measured by ELISA (eBioscience, CA, USA).

### Flow cytometry

Monoclonal antibodies with the following specificities were used in this study: F4/80 (APC), Ly6C (FITC), CD45 (eFluor 450), CD11b (PE-Cy7 or PerCPCy5.5), MHC II (PerCP eFluor 710), CD3 (Alexa Fluor 700), CD4 (Alexa Fluor 605NC), Ly6G (PE), Ter119 (PE), B220 (PE), NK1.1 (PE), CCR2 (PE), IL6 (PE) or TNF (PECy7). Blood and single cell suspensions of lung were stained according to previously published procedures [75]. Total cell counts were determined using CountBright Absolute Counting Beads (Life Technologies). To attain a single-cell suspension of mouse lung tissue, half a lung was collected from each *S*. *pneumoniae*-colonized mouse and kept on ice. Immediately following, each lung was mechanically dissociated and enzymatically degraded using a Miltenyi Biotec Lung Dissociation Kit (Cat#: 130-095-927) along with the gentleMACS Octo-Dissociator with Heaters (Cat#: 130-096-427). Following dissociation as per protocol, cell suspensions were filtered (70 μM cell filter) and centrifuged at 300 x g for 10 min. Subsequently, single-cell suspensions were re-suspended in phosphate-buffered saline & processed for flow cytometry. A gating strategy for distinguishing Ly6C^high^ and Ly6C^low^ monocytes is presented in S3 Fig.

### Cytokine administration

100 nM of recombinant murine CCL2 (endotoxin-free, eBioscience) was diluted in sterile saline and administered intraperitoneally. Recruited cells were isolated via peritoneal lavage and quantitated using flow cytometry. Murine recombinant TNF (eBioscience) diluted in sterile saline was administered intraperitoneally every other day for 3 weeks at a dose of 5 ng per gram of body weight. Adalimumab (HUMIRA, Abbott Laboratories), a humanized anti-TNF antibody, or the human IgG isotype control diluted in sterile saline were administered intraperitoneally at a dose of 50 ng per gram of body weight for a period of 3 weeks.

### Ly6C^high^ monocyte depletion

FITC Fluoresbrite 500 nm carboxylated polsytrene microparticles (PS-MPs) were obtained from Polysciences. PS-MPs were injected via tail vein at 4 x 10^9^ particles in 200 μl as previously described[50]. Monocyte depletion was confirmed by flow cytometry.

### Measurement of cytokine production

Serum TNF and CCL2 was measured using high-sensitivity ELISA as per manufacturer's instructions (Meso Scale Discovery). For quantitative PCR analysis, RNA Lysis Buffer (Qiagen) was used to collect nasopharyngeal RNA via nasal lavage. RNA was extracted using an RNAqueous Micro Kit (Ambion), reverse-transcribed to cDNA using M-MULV reverse transcriptase (New England Biolabs) and qPCR was performed using GoTaq qPCR Master Mix (Promega, WI, USA) and the ABI 7900HT Fast Real-time PCR System (Applied Biosystems, CA, USA) all to manufacturer’s instructions. Cycle threshold (Ct) values relative to the internal reference dye were transformed by standard curve, followed by normalization to the housekeeping gene GAPDH. Normalized results are presented as relative to an internal calibrator sample.

### Quantitation of monocyte-bound *S*. *pneumoniae*

100*μ*L samples of peripheral blood, were incubated with TRITC-labeled *S*. *pneumoniae* (MOI 20) resuspended in 100μL of complete RPMI at 4°C to allow binding, but not uptake. After 30 min of incubation, cells were stained for flow cytometry. Following RBC lysis (1x 1-step Fix/Lyse Solution eBioscience; ref: 00-5333-57) for 10min, cells were washed 2x with PBS to remove excess stain and non-adherent bacteria, and re-suspended in FACS wash (10% fetal bovine solution in PBS). Flow cytometry was performed and the amount of *S*. *pneumoniae* bound by Ly6C^high^ monocytes was quantitated based on the mean fluorescent intensities of TRITC.

### Administration of anti-TNF *in vivo*

Adalimumab (HUMIRA, Abbott Laboratories), a humanized anti-TNF antibody, or the human IgG isotype control diluted in sterile saline were administered to mice. A dose of 50 ng per gram of body weight was given intraperitoneally in a volume of 200 μl every other day, for a period of 3 weeks to young and old WT mice.

### Statistics

Unless otherwise mentioned in the figure legend, statistical significance was determined by two-tailed Mann-Whitney-Wilcoxon tests, one-way analysis of variance or two-way analysis of variance with Fischer’s LSD post-tests where appropriate.

## Supporting Information

S1 Fig Age is characterized by myeloid skewing in mice. (A) Although total leukocyte numbers were not altered with age, there was a skewing towards cells of myeloid lineage, with increases in the total numbers of monocytes and neutrophils, and a decrease in the total number of T cells in the circulation. (B) The number of bone marrow-derived precursor cells capable of differentiating into macrophages following M-CSF stimulation was increased in old mice relative to young mice. (C) With age, bone marrow-derived precursors differentiating into macrophages ex vivo express heightened CCR2 levels during an intermediate stage of the differentiation process. This is in contrast to precursors from young mice, which do not express peak CCR2 levels until the end of the differentiation process. (D) There were no differences in Ly6C^low^ monocyte levels in the circulation in old TNF KO mice. (E) CCR2 levels were significantly higher on Ly6C^high^ monocytes rather than Ly6C^low^ monocytes. Statistical significance was determined by two-tailed Mann-Whitney-Wilcoxon test, one-way ANOVA or two-way ANOVA with Fisher's LSD post-test where appropriate. * indicates *p* < .05, ** indicates *p* < 0.005, *** indicates *p* < 0.0005 and **** indicates p < 0.00005.
(TIF)

S2 Fig Injection with polystryene microparticles (PS-MP) reduces the percentage of Ly6C^high^ monocytes. Mice (n = 7-10/group) were injected with PS-MP day -1, 0, +1, +4and +6 during colonization with *S*. *pneumoniae*. Injection of PS-MP reduces in the proportion of Ly6Chigh monocytes in the (A)circulation and (B) lungs of old mice during S. pneumoniae colonization. Consequently the proportion of Ly6C^low^ monocytes in the (C) circulation and (D) lungs increases, while there is no effect on (E) circulating neutrophils. (F) Reduction of circulating myeloid cells using an anti-Gr-1 antibody also reduces the bacterial load in the nasal wash of old mice. (*,p<0.05, **,p<0.005, ***, p<0.001 one-way ANOVA with uncorrected Fisher's LSD). Mice that reached endpoint prior to day 7 were not included in the analysis.
(EPS)

S3 Fig Flow cytometry gating strategy for Ly6C^high/low^ monocytes. To gate on Ly6C^high^ monocytes (circulating & lung-infiltrating), first A) CD45+ cells (leukocytes) are gated upon. Subsequently, a B) width gate is created to exclude cell aggregates, and C) CD11b+ cells are selected. Using this population, cells can be divided into D) neutrophils and non-neutrophil using SSC and Ly6C surface expression. E) Monocytes are gated upon as Ly6C^+^/SSC^low^ cells, and those that are F) Ly6C^high^ would be defined as Ly6C^high^ monocytes. Using a dump gate positive for NK1.1, CD19, and CD3, it is apparent that no NK cells, B cells or T cells are found in this population. Isotype controls were used for all experiments.
(TIF)
