# Supplementary figures and images for: TNF Drives Monocyte Dysfunction with Age and Results in Impaired Anti-pneumococcal Immunity
Source: PLoS Pathog. 2016 Jan 14;12(1):e1005368. doi: 10.1371/journal.ppat.1005368 (PMC4713203; doi:10.1371/journal.ppat.1005368)

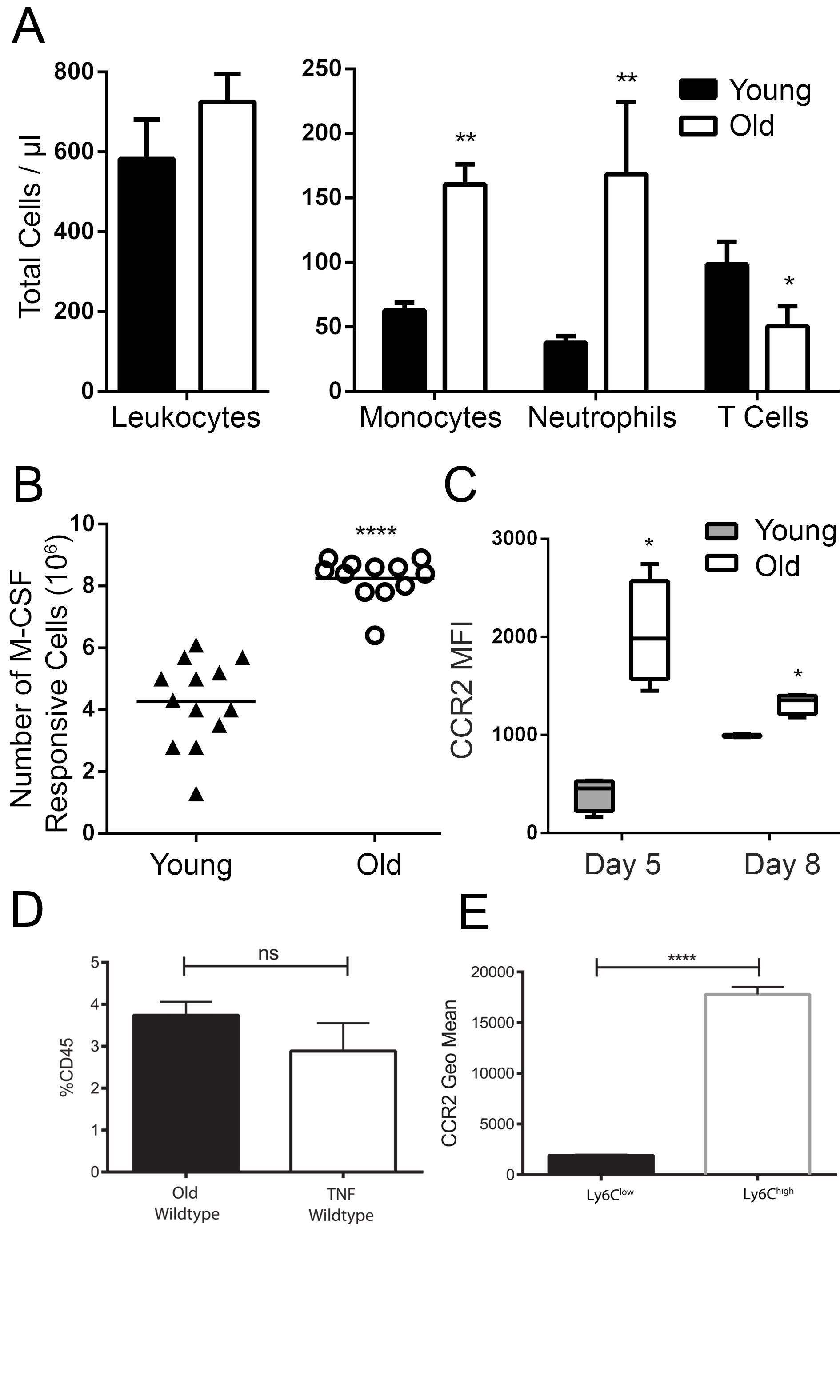

Supplement: S1 Fig — (A) Although total leukocyte numbers were not altered with age, there was a skewing towards cells of myeloid lineage, with increases in the total numbers of monocytes and neutrophils, and a decrease in the total number of T cells in the circulation. (B) The number of bone marrow-derived precursor cells capable of differentiating into macrophages following M-CSF stimulation was increased in old mice relative to young mice. (C) With age, bone marrow-derived precursors differentiating into macrophages ex vivo express heightened CCR2 levels during an intermediate stage of the differentiation process. This is in contrast to precursors from young mice, which do not express peak CCR2 levels until the end of the differentiation process. (D) There were no differences in Ly6Clow monocyte levels in the circulation in old TNF KO mice. (E) CCR2 levels were significantly higher on Ly6Chigh monocytes rather than Ly6Clow monocytes. Statistical significance was determined by two-tailed Mann-Whitney-Wilcoxon test, one-way ANOVA or two-way ANOVA with Fisher's LSD post-test where appropriate. * indicates p < .05, ** indicates p < 0.005, *** indicates p < 0.0005 and **** indicates p < 0.00005. (TIF) [file ppat.1005368.s001.tif]

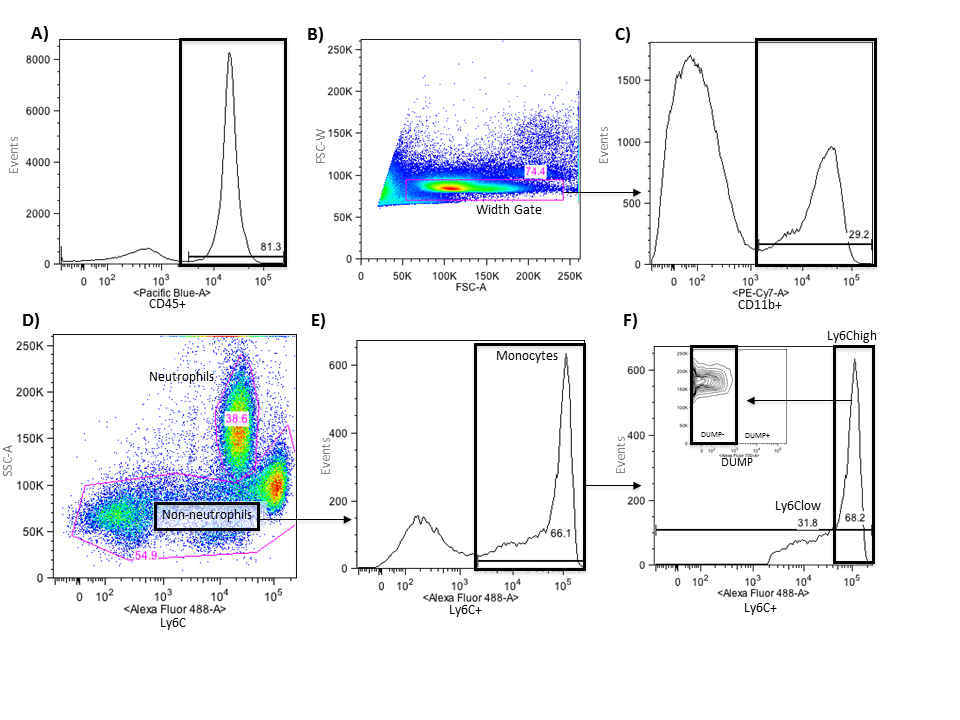

Supplement: S3 Fig — To gate on Ly6Chigh monocytes (circulating & lung-infiltrating), first A) CD45+ cells (leukocytes) are gated upon. Subsequently, a B) width gate is created to exclude cell aggregates, and C) CD11b+ cells are selected. Using this population, cells can be divided into D) neutrophils and non-neutrophil using SSC and Ly6C surface expression. E) Monocytes are gated upon as Ly6C+/SSClow cells, and those that are F) Ly6Chigh would be defined as Ly6Chigh monocytes. Using a dump gate positive for NK1.1, CD19, and CD3, it is apparent that no NK cells, B cells or T cells are found in this population. Isotype controls were used for all experiments. (TIF) [file ppat.1005368.s003.tif]
